# Could COVID-19 be contained in poor populations by herd immunity rather than by strategies designed for affluent societies or potential vaccine(s)?

**DOI:** 10.1080/16549716.2020.1863129

**Published:** 2021-01-04

**Authors:** Andreas Ruppel, Mohammad Ibrahim Halim, Rhondemo Kikon, Nouh S. Mohamed, Mohammad Reza Saebipour

**Affiliations:** a Institute of Global Health, University of Heidelberg, Heidelberg, Germany; b Emergency Response and Covid-19 Directorate, Ministry of Public Health, Kabul, Afghanistan; c Community Health Initiative, Dimapur, India; d Nile University, Khartum, Sudan; e Department of Anatomy, School of Medicine, Birjand University of Medical Sciences, Birjand, Iran

**Keywords:** Covid-19, control, emergencies, inappropriateness, WASH-program, palliative care

## Preamble

Current COVID-19 control strategies are based on social distancing, hygiene, contact tracing and expected vaccine(s). However, these are not suitable for poor and marginalized people living in miserable, unhygienic environments. We hypothesize that among such people the virus will currently spread without restraint and herd immunity may develop before the advent of a vaccine to them. We suggest that improving health and hygiene provision for the poor is necessary *now* to later enable COVID-19 control.

## Current COVID-19 control strategies increase problems of the poor

From the beginning of the COVID-19 pandemic, control efforts focused on social distancing, hygiene, wearing masks and lockdowns as recommended by WHO [1]. These measures were implemented in most countries with local adaptations by many, if not most, governments. The aim was and is to prevent a rapid spread of the virus and the overburdening of health systems, in particular hospitals, with the anticipated needs for intensive care of corona patients with severe and life-threatening conditions. The measures were meant to keep infections at a low incidence to prevent deaths and morbidity, particularly among high-risk populations such as the elderly and people already having additional health risks [2]. The majority of infected younger people remains free of symptoms or suffers transient illness and deaths are comparatively rare in this sector of the population.

To maintain these control efforts massive financial inputs are necessary to mitigate the economic losses due to lockdown. Affluent countries, including Germany, created these funds in the hope that the advent of a vaccine would block transmission and society could return back to ‘normal’. Many less affluent countries (low- and middle-income countries, LMICs) followed this strategy, including lockdowns, as exemplified by Sudan [3], although financial support for people losing work or going bankrupt are barely or not available. The expectation of a vaccine seemed to justify copying measures from the affluent countries. However, it soon became evident that the poorer populations suffered disproportionately by lockdowns. People protested ‘we will die from hunger before we die from corona’ [4,5]. Moreover, the lockdowns caused and still cause break down of health services for the poor, for example HIV, tuberculosis and malaria [6] as well as mother and child care services [7] even where such services had been functional before the advent of corona. Myanmar is an example: ‘[…] in responding to COVID-19, Myanmar faces the challenge of finding – and constantly recalibrating – a balance between restrictive public health measures and the other basic needs of Myanmar’s communities, especially their most vulnerable segments’ [8]. In a world, in which at least half of the global population did not have full coverage of essential health services even before COVID-19 [9], the effects of the control measures introduced in high-income countries (HICs) and which benefit also the affluent quintiles in LMICs, disproportionally stress the poorest, exploited and trafficked persons in many nations [10].

## Among the poor, the virus may spread un-recognized

In the absence of accessible health care for the poor, the corona virus can be expected to spread freely: if you have no home, you cannot go home during a lockdown; if you have no clean water, you cannot follow hygiene measures; if you live as a whole family in one room with neighbours next door under the same conditions, social distancing is simply not possible. The virus is bound to spread rapidly in a crowded environment, particularly so, where targeted tracing and quarantining of contact persons is unrealistic. Thus, even when a whole district of a location or a slum area is locked down, the rapid spread of the virus is inevitable within this population. When testing a person for corona is relatively expensive compared to the annual health expenditure per person, testing remains a privilege for the rich, but an illusion for the poor. As an example, in Afghanistan the per capita annual health expenditure in 2017 was 81 US$ [11] and the COVID-19 test costs are 100$ [12]. By inference, where incidence and prevalence are calculated from reported national test-positive cases, these numbers are very unlikely to represent the reality in geographically remote or poor sectors of the populations (see below).

The poor, living below 1 US$ per day or below national poverty lines represent a large part of the national populations in several countries: for example, in Afghanistan, more than half of the population live with less than 1$ per day [13]; in Tajikistan 26% live under the national poverty line (2019); in Indonesia, despite recent enormous gains, this applies to 10% (2018). The percent living under national poverty lines reached 38% in Zimbabwe (2019), 47% in Sudan (2009), and one or two thirds of populations in a series of other countries (all data from the World Bank [14];). Current data for Sudan were 65% given by Sudan´s Minister of Finance and economic planning [15]. In these situations, numbers of the national test-confirmed COVID-19 cases will be low and not representative of the whole population, because substantial numbers of the poor will not have been tested.

This hypothesis appears to be supported when the official corona cases reported to WHO (as of 12 October 2020) are seen in relation to the nations´ population and number of cumulative tests per 1000 (Table 1). The percentage of reported cases is below 1% among all populations.

**Table 1.** official corona cases reported to WHO (as of 12 October 2020) in relation to the nations’ population and number of cumulative tests per 1000

Selected Country	Confirmed cases (WHO, 12 October 2020)	Population Million (World Bank 2019)	Calculated Cases per population (%)	Cumulative Tests per 1000 (8 October 2020 (Our world in data)
**Africa**				
Nigeria	60,000	201	0.03%	2.68
Sudan	13,700	42.8	0.03%	n.a.
Zimbabwe	8,000	14.6	0.06	8.44
Kenya	41,000	52,6	0.07%	10.69
Zambia	15,000	17.7	0.08	10.12
Ghana	47,000	30.4	0.1%	16.02
**Asia**				
Indonesia	333,000	210	0.12%	8.43
Pakistan	319,000	217	0.15%	17.59
Philippines	339,000	108	0.31%	35.48
India	7,120,000	1,388	0.52%	63.68
Iran	504,000	82.8	0.61%	51.34
**Europe**				
Germany	323,000	83.6	0.30%	216.39
Italy	349,000	. 60.4	0.58%	125.57
France	691,000	65.3	0.10%	n.a.
UK	590,000	67.9	0.88%	337.90

These reported numbers of infected people must be seen in relation to the number of people tested per 1000 population. Where `tested´ numbers are very low, as in Nigeria and Indonesia, they can barely represent the whole country, even less be representative for the national poor populations. The graph ‘Total COVID-19 tests per 1,000 people’ which is available from ‘Our world in Data’ illustrates that this limitation is true for many nations. However, within-country differences give a different picture.

## Within-nations reported tests may not include the poor

In Sudan, an analysis allows important conclusions from passive testing [16]: more than 7200 cases were confirmed in the Capital Khartoum with a case-fatality-rate (CFR) of 3.8%; in various provinces far fewer cases were confirmed, but the CFR was considerably higher: Gazera 955 tests (15.3%), Red Sea 182 (20.9%), North Darfur 142 (31.7%), and Central Darfur 5 (60%). These numbers reflect within-county differences of testing by the health system. The authors conclude: ‘A much higher number of unrecognized cases is extremely probable because of the restricted number of tests, a public reluctance to report infections and, perhaps worse, attitudes of denial’. Overall the testing strategies in Sudan showed 55% positive cases nation-wide and the authors conclude in their publication of August 2020: ‘This high positivity rate (55%) indicates that Sudan is not testing widely enough to find all cases’ [16]. In early October, the reported positive cases were 13.700 (Table 1), but the number of daily reported positive cases had dropped considerably from the peak of June the 16^th^ (520 daily cases) to 0 cases on October the 16^th^ (see: Our World in Data).

Similar pictures of within-nation differences emerge from population-based sero-epidemiological studies. In Afghanistan, a nation-wide survey by the Ministry of Health [17] reported in July 2020 ‘the total proportion of COVID-19 positive infection … was 31.5% for Afghanistan’. Rural areas had 31.7% (CI 26.5–37.4) of positive cases and urban areas 42.3% (35.7–49.2), where `positive´ meant the results of a COVID-19-IgG/IgM Rapid Test Cassette for IgG and IgM antibodies. The authors raised the concern, “if the preventive interventions (in a very bad scenario) are not considered, the peak of COVID-19 was predicted to be in June 2020, and COVID-19 will infect an estimated 69.6% of the population … by end 2020“.

In India, a sero-prevalence survey in 22 states reported for the beginning of May that 0.73% of the 28,000 participants were positive [18]. By June 2020, 23.48% of 21,387 serum samples collected across 11 districts of Delhi had antibodies against COVID [19]. By July 2020, 57% of Mumbai slum dwellers were sero-positive as opposed to 16% in the more affluent areas [20]. In Pune, another hotspot in India, the average sero-prevalence in five high-incidence parts of the city was 51.7% with 65.4% in one part. Those living in hutments had 62% compared to those in bungalows with 43.9%. By size of residence, those living with less than 150 sq ft had 59.6% compared to those with over 500 sq ft and 34.6% [21]. Thus, prevalence appears to be higher in the low social strata compared to the better-off.

Iran is an interesting example of a populous country (ca 82 million), where strict containment and mass quarantine regulations have not been implemented, but where the geographical spread of the virus in the early weeks of the epidemic was documented [22]. The Iranian President was cited by Reuters on 18 July 2020 stating that ‘some 25 million may have been infected with coronavirus’ and this corresponds to more than 30% of Iran’s 82 million population [23]. On October the 22^nd^, the Ministry of Health reported that ‘4,628,866 tests have been carried out’ with “the total number of people tested positive in Iran has risen to 550,757 [24]. The numbers in both of these reports given for the infections refer, respectively, to an extrapolation from tests to the whole population, and the direct number of performed tests. This distinction is relevant to the interpretation of different types of reports. On 29 October the Deputy Minister of Health announced in an interview in Persian Language with the Iran national TV that an estimated 35 million Iranians, equivalent to 40% of the country’s population were infected with corona. The population apparently proceeds versus development of herd immunity.

## Among the poor, Covid-19 prevalence may approach values associated with herd immunity

For herd immunity, ‘there is little evidence to suggest that the spread of SARS-CoV-2 might stop naturally before at least 50% of the population has become immune’ [25]. By analyzing data from Europe and countries affected early during the COVID-19 epidemic, such as Spain and Italy, the paper suggests that the nationwide prevalence of antibodies varies between 1% and 10%, with peaks around 10–15% in heavily affected urban areas. By contrast, some of the above reported prevalence numbers among poor societies may be interpreted to be close to the 50% of serologically positive necessary to reach herd immunity. Since COVID-19 spreads quickly in the absence of efficient control measures, the ‘real’ case numbers (as opposed to passively detected ‘test-positive’ cases) should still increase rapidly in the near future among the poor, where access to health services is low or absent and the official control measures are unlikely to work out due to the living conditions. Calculating the excess mortality compared to the same period of the previous year can be used as a tool to estimate mortality caused by COVID-19 [26]. The authors suggest that ‘It can be challenging to differentiate between people who died of COVID-19 and those who were infected but died from unrelated causes’. This analysis stated that the excess death toll is not reflected in official COVID-19 death statistics and reported the case of Peru, where 74% of the excess deaths are not explained by reported COVID-19 deaths. This is the bad news.

The good news is that these poor populations may have reached or will reach herd immunity soon and, in any case, much sooner than in affluent societies with efficient COVID-19 control. Consequently, a ‘second wave’, which is much feared in affluent societies [25] appears to be unlikely for populations where herd immunity will be reached after their currently progressing ‘first wave’.

Herd immunity may be reached unnoticed in poor societies for several reasons. (i) These populations had long been neglected (lack of access to essential health care [9]), and their suffering from other conditions has been insufficiently addressed in the past. People got sick and may have died for many years officially unnoticed. With corona, it would be unrealistic to expect the situation to change within a few weeks or months. (ii) The populations in the countries mentioned above are younger than in HICs or the affluent sections of LMICs. Corona infections remain largely asymptomatic among children and the young, although asymptomatic carriers of the virus may be infective to others. Thus, in the poor population sectors, infections are possibly `silent´. This hypothesis is supported by the numbers of excess deaths reported during the pandemic compared to mean death numbers in pre-corona years [26]. (iii) The poor have always suffered from various infectious as well as non-communicable diseases, and COVID-19 cases may simply remain unnoticed against this background of the already existing double burden of diseases. (iv) The poor run much higher risks than the rich of dying from prevalent miserable conditions of poor hygiene, lack of drugs, inability to pay for out-of-pocket expenses for medical treatment. Thus, those surviving these harsh lives may have fewer disease conditions which contribute to deaths among the elderly rich. (v) Lastly, it seems unrealistic that the poor marginalized will rapidly benefit from a vaccine. The COVAX initiative announced on September 3rd its initial aim to have 2 billion doses available by the end of 2021. This is certainly a laudable aim, however, until this is reached COVID-19 is likely to continue to spread among those already suffering now from extreme misery [4,5].

Evidence that many corona deaths are not noted by the official counts was detailed by The New York Times [27] by comparing the excess deaths above normal observed in 2020 with the reported COVID-19 deaths. The discrepancies between these numbers represent the deaths due to corona but not recorded as such. According to these analyses, world-wide there are now (update of November 27th) 412,000 missing deaths. To cite a few examples, excess deaths were prominent in Peru and several other nations of South America during the first part of the year, but are decreasing during the second. Jakarta is a megacity where excess mortality persists. Interestingly, Mumbai experienced a prominent peak, but death rates have now returned to normal. It appears that the coronavirus outbreak may now be limited in Mumbai (for whichever reason). Numbers might be related to the development of the disease in the slums. About 900 million people world-wide live in slums [28]. These are situations, where the virus will spread `undisturbed´.

In conclusion, herd immunity against COVID-19 may have been reached or is likely to be reached in a not too far future among the disadvantaged and the poor, including the 1.8 billion people around the world who are homeless or live without adequate shelter [28]. For them, a ‘second wave’ may not be a real threat, however, they will have already paid a huge price in terms of health, deaths and finances – mostly overlooked by decision makers. Until a vaccine becomes available, it seems unlikely that the poor will be the first to benefit from this preventive tool [29]. Among them, the virus will have a prolonged time to spread. We are afraid that this is the current reality for the marginalized poor. In fact, after Pfizer and Biotech announced the success of their vaccine on November 9^th^, the European Union bought two days later 300 million doses; still a few days later Curevac announced its intention to produce up to 300 million doses of its COVID-19 vaccine in 2021 and up to 600 million in 2022. The WHO has warned that use of the principle of ‘herd immunity’ to stem the COVID-19 pandemic is ‘unethical’ and ‘not an option’ [30]. We hope that warning will be followed by a political will to target not only the better-off populations but also the poor.

## How could public health professionals help?

What could help the poor – at relatively low cost compared to the economic investments into saving the rich populations from corona and to the costs invested into vaccines? As public health professionals, we should advocate for creating living situations for the poor that are essential for corona control: decent housing, provision of clean water and hygiene [31]. This would simultaneously prevent other causes of child and adult morbidity and mortality, e.g. diarrhea and neglected tropical diseases. We should be a strong voice to advocate for essential health coverage [9] by using corona as the trigger to end the political neglect of the poor and promote the first Sustainable Development Goal to end poverty. We suspect that implementing such immediate pro-poor actions compares favorably with the “Gavi COVAX Advance Market Commitment“ (October the 13^th^) statement that ‘it will be important to get this US$ 2 billion funding secured by the end of 2020 to ensure that agreements can be put in place’. We might advocate the introduction of rapid palliative care for those who are threatened by corona-death, and at the same time for those dying from other diseases for which they lack the out-of-pocket money to be cured. As public health professionals, we should promote inter-sectoral approaches to integrate corona services with other locally or nationally established programs. We may worry less for a `second corona wave´ among the poor but use global attention to the pandemic as leverage to care also for the multitude of their other serious health issues.

This manuscript draws on presentations and discussions during an internet-based workshop “Implementation of health promotion in complex social and political situations” (September 5 to 13, 2020). The workshop was organized by AR on behalf of the Institute of Global Health, University of Heidelberg, Germany. It was financially supported by the German Academic Exchange Service (DAAD) with funds provided by the German Federal Ministry for Economic Cooperation and Development. Participants were from 24 nations. We are grateful to Dr. Ann Bartlett, London, for correcting the English of the final manuscript.

## Paper context

Current COVID-19 control strategies are based on social distancing, hygiene, contact tracing and expected vaccine(s). However, these are not suitable for poor and marginalized people living in miserable, unhygienic environments. We hypothesize that among such people the virus will currently spread without restraint and herd immunity may develop before the advent of a vaccine to them. We suggest that improving health and hygiene provision for the poor is necessary now to later enable COVID-19 control.
